# From Treatment Discontinuation to Aggressive Recurrence: A Cautionary Case Report of Rapid Progression to Invasive Carcinoma Following Torimifene‐Induced Hepatotoxicity and Drug Withdrawal in a Patient With Completely Resected Intraductal Papillary Carcinoma

**DOI:** 10.1002/ccr3.73061

**Published:** 2026-07-28

**Authors:** Lanya Yu, Zhe He, Guibin Qiao

**Affiliations:** ^1^ Department of General Surgery General Hospital of Southern Theater Command Guangzhou China; ^2^ Department of Thoracic Surgery Zhujiang Hospital of Southern Medical University Guangzhou China

**Keywords:** case report, drug‐induced liver injury, intraductal papillary carcinoma, invasive recurrence, toremifene, treatment adherence

## Abstract

Even ‘low‐risk’ intraductal papillary carcinoma can progress rapidly to invasive disease following unplanned discontinuation of adjuvant endocrine therapy, as illustrated by this case where hepatotoxicity led to treatment cessation and early recurrence. This underscores the critical need for proactive toxicity management, patient education, and consideration of enhanced surveillance for high‐risk patients who interrupt therapy.

## 1 Introduction

Intraductal Papillary Carcinoma (IPC), a distinct subtype of ductal carcinoma in situ (DCIS), accounts for 1%–2% of all breast cancers [1]. Characterized by a papillary growth pattern, it typically exhibits low‐to‐intermediate nuclear grade, high hormone receptor positivity, and a significantly better prognosis than common invasive carcinomas, with 10 year disease‐free survival rates often exceeding 90% [2]. Standard treatment involves complete surgical excision, often supplemented with endocrine therapy for hormone receptor‐positive patients to reduce ipsilateral and contralateral breast cancer risks [3].

While IPC is considered low‐risk, tumor cells in DCIS possess the potential to progress to invasive breast cancer [4]. This report presents a cautionary case of an ER‐positive IPC with a high Ki‐67 index that recurred as invasive carcinoma on the chest wall shortly after discontinuation of adjuvant toremifene due to drug‐induced liver injury. We explore the implications of treatment interruption, tumor biology, and strategies for managing high‐risk DCIS/IPC patients.

## 2 Case History

**FIGURE 1:**
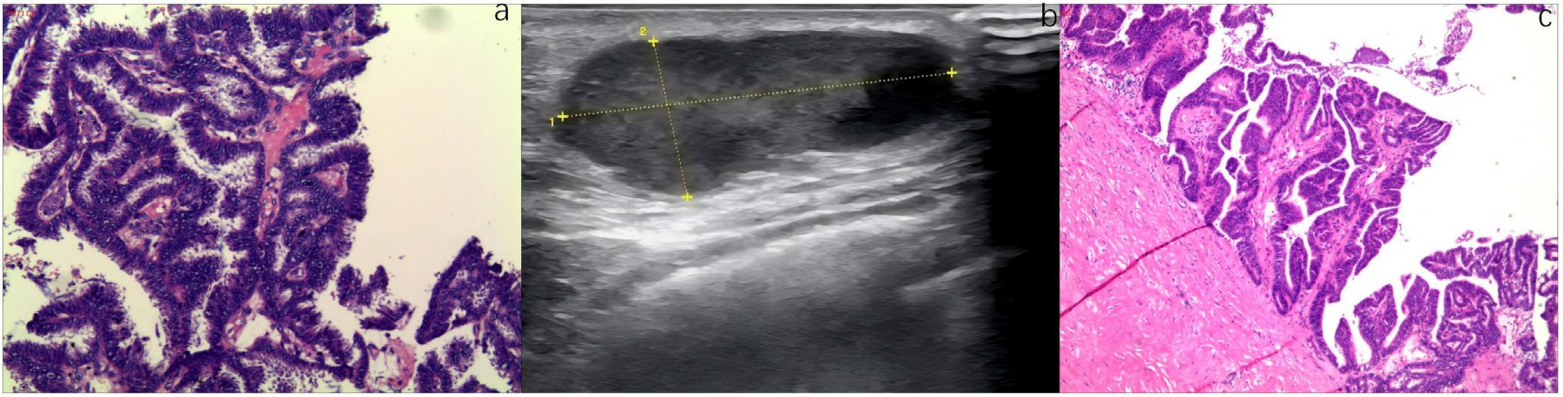
(a) The primary lesion shows papillary hyperplasia of the mammary ductal epithelium. Immunohistochemical staining for CK5/6, P63, and CD10 is negative for myoepithelial cells. High‐power magnification. (b) A hypoechoic lesion was detected in the right chest wall, approximately 3.2 × 1.4 cm, with clear boundaries and regular shape. The internal echogenicity was inhomogeneous, and a liquid dark area was visible. (c) The tumor invades the muscle, and the papilla and surrounding area lack myoepithelial cells. Low magnification.

In February 2023, a 41‐year‐old woman with no family history of breast cancer presented with a progressively enlarging, painful right breast mass over 2 years. Physical examination revealed a firm mass in the upper outer quadrant. Ultrasound identified a mixed‐echo nodule (BI‐RADS 4C). Excisional biopsy confirmed IPC (Figure 1a). Immunohistochemistry (IHC) showed ER (80%+), PR (80%+), CerbB‐2 (−), and Ki‐67 (20%+).

The patient underwent mastectomy in February 2023 and initiated adjuvant toremifene (60 mg/day). After 19 months (September 2024), she developed fatigue and anorexia, with markedly elevated ALT (ALT value in 151 U/L) attributed to toremifene‐induced liver injury, leading to self‐discontinuation (HBV DNA: [300 IU/mL], HBeAg: [negative], no prophylactic antiviral therapy given).

Two months post‐cessation (November 2024), she palpated a pea‐sized, firm subcutaneous nodule in the right chest wall. Ultrasound imaging (Figure 1b) confirmed a 3.2 cm × 1.4 cm hypoechoic nodule in the subcutaneous tissue. IHC of the recurrence showed ER (95%+), PR (95%+), CerbB‐2 (−), and Ki‐67 (20%+). Delayed biopsy revealed invasive papillary carcinoma infiltrating striated muscle (Figure 1c). Staging (bone scan, CT) showed no distant metastasis.

A multimodal regimen was initiated: 6 cycles of TC (docetaxel + cyclophosphamide), radical radiotherapy to the chest wall and regional nodes, followed by intensified endocrine therapy (goserelin + anastrozole + abemaciclib). The patient remains stable (last follow‐up: May 2026, 8 months post‐recurrence treatment) under follow‐up.

### 2.1 Methods: Differential Diagnosis, Investigations and Treatment, Treatment Course

The patient underwent a right total mastectomy with sentinel lymph node biopsy in February 2023. Final pathology confirmed complete resection with negative margins and no metastasis in sentinel lymph nodes (0/3). Given the hormone receptor‐positive status, adjuvant endocrine therapy with oral toremifene (60 mg/day) was initiated.

### 2.2 Adverse Event and Treatment Discontinuation

After approximately 19 months of therapy (September 2024), the patient developed fatigue and anorexia. Laboratory investigation revealed a markedly elevated alanine aminotransferase (ALT) level (151 U/L). Other common causes of liver injury were excluded. The patient had a history of hepatitis B but presented with normal pre‐treatment transaminase levels. The acute liver injury during treatment showed a clear temporal relationship with toremifene administration and was therefore more likely attributable to the drug's hepatotoxicity; however, the pre‐existing hepatitis B history may have been a potential predisposing factor (HBV DNA: [300 IU/mL], HBeAg: [negative], no prophylactic antiviral therapy given). This led the patient to self‐discontinue the medication.

### 2.3 Diagnosis of Recurrence

Two months post‐cessation (November 2024), the patient palpated a pea‐sized, firm nodule in the right chest wall. Ultrasound imaging at discovery demonstrated a hypoechoic lesion measuring 3.2 × 1.4 cm (Figure 1b). Biopsy was delayed until May 2025 due to personal reasons. Histology confirmed invasive papillary carcinoma infiltrating striated muscle (Figure 1c). IHC of the recurrence showed ER (95%+), PR (95%+), HER2 (−), and Ki‐67 (20%). Staging via bone scan and CT chest/abdomen revealed no distant metastasis.

### 2.4 Treatment for Recurrence

A multidisciplinary tumor board recommended a multimodal approach: four cycles of TC chemotherapy (docetaxel/cyclophosphamide), followed by radical radiotherapy to the chest wall and regional nodes. Subsequently, intensified endocrine therapy was started, comprising ovarian function suppression (goserelin), an aromatase inhibitor (anastrozole), and a CDK4/6 inhibitor (abemaciclib).

## 3 Follow‐Up

The patient tolerated the combined modality treatment for recurrence. At the most recent follow‐up in May 2026 (8 months post‐treatment), she remains clinically stable with no evidence of disease progression.

## 4 Discussion

Regarding hepatotoxicity, available original studies suggest that clinically significant liver injury from toremifene is uncommon but not negligible. Hamada et al. reported computed tomography‐detected fatty liver in 4 of 52 patients (7.7%) receiving adjuvant toremifene, including 1 patient with biopsy‐proven non‐alcoholic steatohepatitis [5]. In a larger retrospective study, Yang et al. found that 45 of 618 SERM‐treated patients with normal baseline ALT developed ALT elevation during treatment, and 92.9% normalized after discontinuation [6]. Song et al. further showed that the 3 year incidence of new‐onset fatty liver was lower with toremifene than with tamoxifen (17.53% vs. 25.97%), suggesting that toremifene may be comparatively less hepatotoxic, although not free of hepatic risk [7]. More recently, Wang et al. reported no overall deterioration in hepatic function among 597 patients receiving adjuvant tamoxifen or toremifene over serial follow‐up, underscoring that severe clinically apparent hepatotoxicity is rare in routine practice [8]. Nonetheless, rare severe cases, including toremifene‐associated liver failure, have been published [9]. Taken together, the literature supports describing toremifene hepatotoxicity as uncommon, heterogeneous in presentation, and usually reversible after withdrawal, while also justifying continued monitoring during therapy.

This case illustrates an aggressive transformation in a typically indolent disease. The rapid emergence of invasive recurrence shortly after toremifene cessation suggests that treatment interruption may have removed a critical inhibitory signal on residual hormone‐sensitive cells [10, 11]. The high Ki‐67 index in both primary and recurrent tumors indicates a proliferative phenotype, potentially amplifying the risk upon estrogen pathway reactivation.

The late‐onset hepatotoxicity underscores the need for sustained vigilance and patient education regarding side effects throughout endocrine therapy, not just at initiation. For high‐risk patients (e.g., high Ki‐67) who must discontinue standard therapy, our experience argues for considering enhanced surveillance protocols and prompt multidisciplinary evaluation for alternative strategies, such as switching to an ovarian suppression‐based regimen [12].

The primary limitation is the inherent inability of a single case report to prove causation between drug discontinuation and recurrence. The patient's self‐discontinuation and diagnostic delay also limit generalizability but starkly highlight real‐world challenges in adherence and follow‐up.

## 5 Conclusion

The rapid recurrence of invasive carcinoma following toremifene‐induced hepatotoxicity and treatment interruption emphasizes the importance of vigilant monitoring, patient education, and proactive management of endocrine therapy side effects. Enhanced surveillance and timely intervention are recommended for high‐risk IPC patients who experience treatment discontinuation.

## Author Contributions

**Lanya Yu:** investigation, writing – original draft. **Zhe He:** methodology, writing – review and editing. **Guibin Qiao:** project administration, supervision.

## Funding

This work was supported by a grant from Noncommunicable Chronic Diseases‐National Science and Technology Major Project (2024ZD0529400 and 2024ZD0529401).

## Ethics Statement

Written informed consent was obtained from the patient, and the research was approved by the Ethics Committee of The General Hospital of Southern Theater Command on May 19, 2025.

## Data Availability

All raw data and code are available upon request.
